# Ex-PALM: a negative pressure ventilation system for air leak evaluation in *ex vivo* lungs

**DOI:** 10.3389/fbioe.2025.1725254

**Published:** 2026-01-22

**Authors:** Mark Vartazarmian, Alexandre Abid, Rodin Chermat, Maxime Têtu, Luciano Bulgarelli Maqueda, Arman Sarshoghi, Saman Naghieh, Delphine Périé, Moishe Liberman

**Affiliations:** 1 Department of Mechanical Engineering, Institute of Biomedical Engineering, Polytechnique Montréal, Montreal, QC, Canada; 2 TID Laboratory, Centre de Recherche du Centre Hospitalier de l’Université de Montréal (CRCHUM), Montreal, QC, Canada; 3 Centre Hospitalier de l’Université de Montréal (CHUM) Endoscopic Tracheobronchial and Oesophageal Center (CETOC), Division of Thoracic Surgery, University of Montréal, Montréal, QC, Canada

**Keywords:** air leak, *ex vivo*, lung model, cough, negative pressure ventilator, thoracic surgery, tissue mechanics

## Abstract

**Introduction:**

Prolonged air leaks (PAL) are considered to be one of the leading causes of postoperative complications following lung surgery. There are currently no clinically relevant methods for efficiently and systematically evaluating the underlying causes of PAL. Here, we introduce a new intuitive, physiologically-representative system for *ex vivo* negative pressure ventilation of lungs, equipped with PAL-oriented features.

**Methods:**

Reproducibility and system capabilities were assessed using a lung simulation model capable of controlling the effective area of the defect, then validated with *ex vivo* specimens.

**Results:**

Our system enables dynamic control of ventilation using either pressure (PCV) or volume (VCV) targets, with respective standard deviations of ±0.08 cm H2O and ±2.1 mL with moderate air leaks (<1,000 mL/min) and respective standard deviations of ±0.18 cm H2O and ±11 mL with severe air leaks (>1,000 mL/min). Additionally, leak quantification features proved comparable to that of the Thopaz+ (Medela Healthcare, Baar, Switzerland), a standard commercial digital thoracic drainage system, offering sufficient resolution to differentiate among clinically relevant air leaks. In the lower leak ranges (<400 mL/min) across all methods of evaluations, there were no significant differences between measured leak rates. For higher leak ranges, although there remained no significant differences between the Ex-PALM methods evaluated, the *Thopaz* + proved to systematically report lower leak rates values (*Thopaz*+ 420.0 ± 10.0 mL/min vs. PCV-derived 449.0 ± 19.9 mL/min, p < 0.05) and (*Thopaz*+ 1,200.0 + 0.0 mL/min vs. PCV-derived 1,239.7 ± 21.1 mL/min, p < 0.001). Unlike current systems, coughing was predictably replicated using peak pressure targets ranging from 100 to 300 cm H2O with a standard deviation of ±1.30 cm H2O from target. Our system allows extraction of biomechanical parameters at every breath, with theoretically expected pressures matching experimental measurements with a goodness fit value (R2) above 0.95 for the vast majority of breaths.

**Discussion:**

The *Ex vivo* Pulmonary Air Leak Model (Ex-PALM) provides a preclinical PAL testing platform with high translational potential and applications in studying biomechanical mechanisms of PAL and developing intraoperative mitigation strategies.

## 1 Introduction

Lung cancer is the leading cause of cancer-related deaths and the second most common cancer worldwide, with an estimated 2.2 million new cases reported in 2020 (Ferlay et al., 2021). In non-small cell lung cancer (NSCLC), which represents the majority of cases (∼85%), the primary treatment for early-stage lesions is some form of lung resection via minimally invasive surgery (e.g., wedge resection, segmentectomy or lobectomy) (Gridelli et al., 2015; Cowper et al., 2021). However, in 5%–19% of cases, patients are post-operatively diagnosed with prolonged air leaks (PAL), suggesting the presence of one or more channels through which inhaled air may travel directly from the bronchial tree into the pleural space (Attaar et al., 2020). Despite the countless techniques and products devised to mitigate their presence, PAL remain a major source of burden for healthcare systems and patients alike (Liberman et al., 2010; Swanson et al., 2014).

Nevertheless, there is a lack of a reliable and efficient methodology to properly investigate and understand the underlying causes leading to the occurrence of PAL. A major hurdle in this quest can be attributed to the delayed manner in which PAL become apparent; often only emerging when the thorax is closed and the clinicians no longer have access to the region of interest. Whether the issue lies in the need for better equipment to adequately identify and localise the leaks intraoperatively, or in the mechanism delaying the occurrence of the defect leading to a leak, remains unclear. Thus, it is essential to evaluate the lungs over a sufficiently long period of time and under different clinically relevant conditions. Such conditions include sighing and coughing, to properly capture the range of postoperative scenarios a patient is likely to experience (Cerfolio et al., 1998; Cao et al., 2022).

In that regard, *in vivo* clinical trials are resource-intensive, can only be conducted for products at advanced stages of development and certification, and often suffer from inter-patient and inter-clinician variability (Yu and Laohathai, 2022). Conversely, there currently exists no published *in vitro* system capable of adequately emulating the complex structure and tissue composition of the lungs at the appropriate scale. In light of these challenges, there is a growing need for a preclinical model with high translational potential that combines the clinical relevance of *in vivo* with the versatility and experimental flexibility provided by *ex vivo/in vitro* approaches.

The starting point for developing a relevant model that replicates typical conditions to which the lungs are exposed to in the postoperative, spontaneously breathing, non-ventilated setting is the choice of ventilation approach. It appears that favoring the use of negative pressure ventilation (NPV) (Aboelnazar et al., 2018; Grasso et al., 2008), over the more standard positive pressure ventilation seen in standard mechanical ventilators, can be favorable in avoiding unnatural stresses on the lung tissue and in limiting ventilator-induced lung injury. Similar to the actions of the diaphragm and intercostal muscles on the pleural environment, NPV can be achieved in explanted lungs by generating cyclical pressure gradients on the lung parenchyma to induce airflow while maintaining an unobstructed airway.

In recent years, numerous devices have been proposed regarding NPV of animal and human lungs, the most notable being a functioning NPV system capable of quantifying leaks for comparative studies of air leak occurrence across different stapling technologies (Klassen et al., 2018). However, this device requires a liquid interface (i.e., saline) to generate the necessary pressure gradient within the lung enclosure, a cumbersome approach that prevents access to the lung during ventilation. In addition, it remains unclear how the liquid will interact with the leak throughout the experimentation, as it is unnatural for the lungs to be bathed in liquid in the post-operative chest for a prolonged period.

Similarly, Aboelnazar et al. introduced a device as a complement to their *ex vivo* lung perfusion (EVLP) setup, aimed at prolonging lung preservation in the context of transplantation (Aboelnazar et al., 2018). Although promising for extending the range of acceptable lungs for transplantation, this device requires a dual-ventilator system introducing an airway pressure typically absent in passive breathing and was not designed with PAL-oriented regulation or evaluation in mind. Finally, the piston-based devices described by Wong et al. (2017) and Sattari et al. (2020), also lack the PAL-oriented features and capabilities to achieve long-term ventilation on non-leak free lungs.

As such, pre-clinical PAL research is currently hindered by the technical limitations and poor translational potential of currently available *ex vivo* systems. In this study, we developed and validated the *Ex vivo* Pulmonary Air Leak Model (Ex-PALM), a new clinically relevant system for negative pressure ventilation of *ex vivo* lungs, featuring seamless integration of physiological biomechanics and quantitative dynamic analysis. Yielding a more complete and less cumbersome NPV system aimed at accurately studying PAL dynamics and their mitigation, the Ex-PALM system was specifically designed to address current limitations with the following list of requirements: prolonged (>6 h) NPV in normothermic environment, air leak compensation and quantification methodology, physiological coughing capability, real-time evaluation of lung biomechanics, along with compatibility for both healthy and diseased animal and human lungs.

## 2 Materials and methods

### 2.1 Hardware overview

**FIGURE 1:**
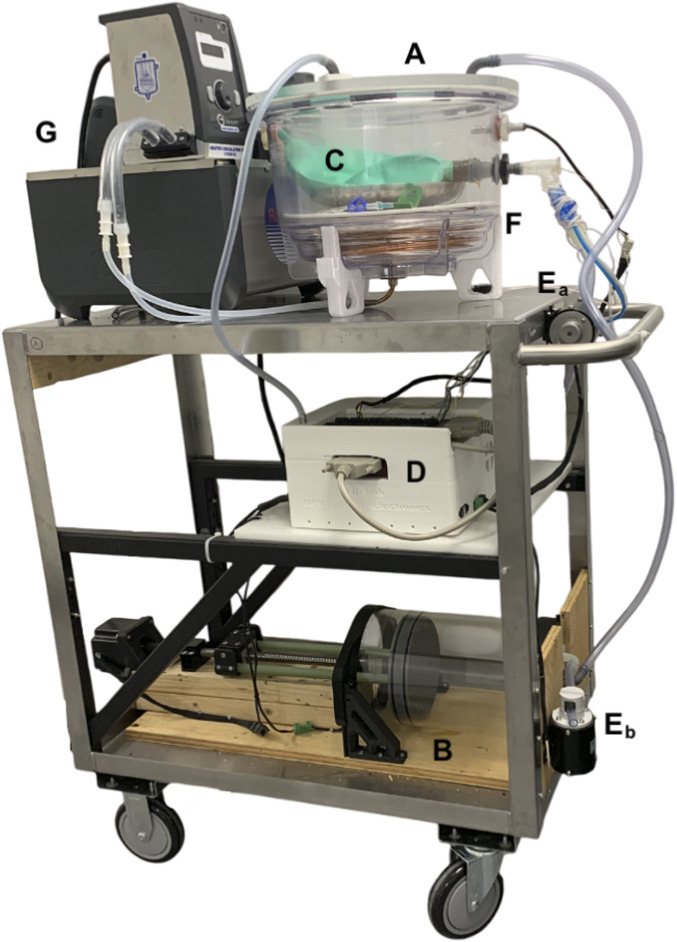
The Ex-PALM consisting of: **(A)** the airtight lung enclosure, **(B)** the motor driven piston, **(C)** a pair of *ex vivo* lungs (double neoprene balloon in this case), **(D)** the electronics box (including sensors, microcontroller, power supply, etc.), **(E**_**a**_ **)** the pinch valve A equivalent to the Epiglottis, **(E**_**b**_ **)** the pinch valve B for purging, **(F)** the copper coil serving as heat exchanger and **(G)** a thermal bath.

Mechanically, the device was designed to utilize both off-the-shelf components and parts fabricated with the use of additive manufacturing processes when additional customisation is necessary. All customised parts were designed using a computer aided design (CAD) software (Fusion 360, Autodesk, CA, United States). The essence of the design lies in the generation of a pressure gradient within an airtight lung enclosure containing the *ex vivo* pair of lungs to be ventilated (Figure 1). This was achieved by the transfer of rotational motion from a stepper motor (3.32 Nm, Nema 24; McMaster, Illinois, United States), controlled using a stepper driver (6627T912: McMaster, Illinois, United States), to a worm gear, which delivers linear motion to a 3D-printed piston head, tightly fitted with O-rings, in an acrylic cast chamber (6.35 mm thick). Knowing the pitch of the worm gear (5 mm/rotation) and the inner diameter of the 8L cylinder (173 mm), the volume variation of the piston for each angular increment can be determined. The output of the piston being connected directly to the airtight lung enclosure above, allows the fluctuation of volume for the humid air interface of the lungs. To ensure accurate conversion of motor angular increments to volume changes, it was calibrated by filling the piston with water and repeatedly expulsing it into a 200 mL medical syringe. Additionally, piston and lung volume management are accomplished with a set of two pinch valves (*EPK-1005-NO-9-E*: Takasago Electric Inc., Aichi, Japan) fixed to the output of the lung (pinch A) and to the output of the piston (pinch B).

When not perfused, the temperature can be controlled by a body of water at the bottom of the lung enclosure, which is never in direct contact with the organ, it is subsequently heated using a thermal bath (170051G-115; ADinstruments, Otago, New Zealand) circulating water through a fully submerged heat exchanger. This design has a double purpose: maintaining a steady state normothermic temperature and generate humidity to mimic the lubricating action of the pleural fluid (keep the surface well hydrated). To compensate for loss caused by poor enclosure insulation, the said body of water is maintained at 15 °C above the target temperature and the lungs are placed on a double-walled aluminum plate providing insulation issuing from the pocket of air enclosed within the aluminum walls. To maintain visibility inside the enclosure during ventilation, the lid is heated using a heated pad. Similarly, to avoid condensation on the surfaces of the flow meter, it is wrapped in a heated cable. For adequate control and monitoring, a temperature and humidity probe (HPP809A031, TE connectivity; Galway, Ireland) is placed in the lung enclosure (T_box_) and a thermocouple (K-type, 5LSC-5SRTC; OMEGA engineering, CT, United States) is inserted in the airway near the main bronchi bifurcation (T_lung_). Enclosure pressure ( $P_{box}$ ) is measured using a pressure transducer (SS302-IP65; Sendo-Sensor, Zhejiang, China) connected to its dedicated port and airway pressure is measured using a differential pressure sensor (MPX5050DP; NXP semiconductors, Eindhoven, Netherlands) at the outlet of the airway. Lung flow and volume are measured using a proximal flowmeter (PN281637; Hamilton Medical Graubünden, Switzerland) in conjunction with a differential pressure sensor (SDP816-125PA; Sensirion, Stäfa, Switzerland).

### 2.2 Software and sensor overview

Aside from the thermal bath and the heated cables, all active interactions with electronics (i.e., sensors, motor and valves) are done through a custom LabVIEW-based program (National Instruments, TX, United States) communicating to a data acquisition module (T7-Pro; LabJack, Colorado, United States) operation at a 140 Hz frequency. In addition to storing all pertinent data in comma-separated values (CSV) files for later post-processing, the user interface of the program generates real-time graphs, displays sensor measurements and allows the user to modify typical ventilatory parameters in accordance with the experimental protocol. Some of the key directly controllable parameters are plateau pressure (P_plat_), residual pressure (P_res_), tidal volume (V_t_), end inspiratory pause (EIP), end expiratory pause (EEP), inspiratory and expiratory ratio (I:E), piston speed (v_p_), piston acceleration (a_p_), peak coughing pressure (P_peak_) and deep inspiration pressure (P_DI_). Other parameters such as breaths per minute (BPM) are said indirectly controllable considering they can be modified using other parameters (e.g., piston speed, EIP, EEP and so on).

All sensor analogue outputs are processed using the manufacturer recommendation to get the proper conversion to digital measurements. As for the lung volume, it is obtained by integrating the airway flow (Q) using a trapezoidal approximation method (Equation 1). The current tidal volume V_t_ is constantly recalculated using the previously measured volume (V_t, i-1_), the current airway flow (Q_i_), the previously measured flow (Q_i-1_) and the intervals ( Δt ) between measurements; about 7 ms.

$$V_{t}=V_{t,i-1}+\frac{Q_{i-1}+Q_{i}}{2}\Delta t$$ (1)

Q was inferred by the pressure variation ΔP_flow_ measured at the inlet and outlet of the dedicated flowmeter. Due to its flexible diaphragm, the flow of gas passing through the perturbation creates a turbulence generating a ΔP_flow_ proportional to Q. Having little information on the exact relationship between Q and $\Delta P_{flow}$ of the device used, a LabVIEW polynomial fit function was employed to infer a cubic polynomial specific to the unit used. Accounting for the accumulation of error regarding the integration and the additional flow related to inhaled air escaping from a lung defect, V_t_ is reset to zero after each expiration.

### 2.3 Control strategy

As Boyle’s Law states, the pressure of a given mass of gas is inversely proportional to its volume at constant temperature (Equation 2). In other words, the product of the pressure (P) and its volume (V) is equal to a constant (cst) (Broaddus, 2016).

P·V=cst (2)

In our case, this implies we can rely on the variation of volume representing the gas interface (V_i_) (i.e., humid air), to generate the pressure gradient necessary for ventilation. Reworking Equation 2 to fit our physical model, we get that the total volume occupied by the gas interface within the sealed area (V_tot_); includes the piston volume (V_p_), the lung volume (V_l_) and the fixed enclosure volume (V_0_) also considered as device dead space not contributing to pressure variation.

$$V_{tot}=V_{p}+V_{l}+V_{0}$$ (3)

Substituting Equation 3 in Equation 2, and considering we take the states at different arbitrary points in time illustrated as *i* and *i-1*:

$$V_{l,i}=\frac{P_{i-1}\left(V_{p,i-1}+V_{l,i-1}+V_{0}\right)}{P_{i}}-V_{p,i}-V_{0}$$ (4)

At first glance, this implies that a decrease in P_i_ or V_p,i_ will lead to an increase of the term on the right in Equation 4. This representation quickly becomes difficult to work with if one considers that the inflation and deflation of the lung depend on the pressure applied to its surface, but the volume it occupies within the lung enclosure also plays a role in the calculation of P_i_. To avoid unnecessary assumptions, this interdependence led to choosing a control strategy based on the accurate knowledge of the motor’s angular position and consequently the V_p_. The different enclosure pressures desired for ventilation are reached by setting upper and lower positional targets the motor must move to. Those targets are then automatically modified at each breath cycle to consistently achieve ventilation in accordance with user input parameters.

For pressure-controlled ventilation (PCV), the main ventilatory parameters input by the user are P_plat_ and P_res_ respectively measured during the end-expiratory pause (EIP) and the end-inspiratory pause (EEP). Thus, to achieve the desired ventilation, after lung recruitment, upper and lower positional targets were empirically determined by inhaling until P_box_ equalled the chosen P_plat_, then exhaling until the chosen P_res_ was reached. This enables the software to translate these ventilatory parameters into angular positions the motor can actually aim for. Similarly, volume-controlled ventilation (VCV) respects the same logic, but uses the tidal volume as its upper target. Instead of targeting a residual volume as the lower target, the user input P_res_ was retained due to the low reliability of the volume measurement.

### 2.4 Leak compensation

Contrary to common turbine-driven mechanical ventilators, the use of a piston, although simplifying some aspects of the system, also brings inherent limitations requiring careful considerations. Most notably, having to work around a fixed operational volume of 7,000 mL; when dealing with any form of air leakage, this can quickly become a concern. Whether they stem from the system itself, a procedure conducted on the organ in use or for example, the deteriorating nature of an explanted organ, improper compensation of leaks will result in an inability to maintain stable ventilation for long durations of time. To cope with this, a piston volume management strategy was developed.

**FIGURE 2:**
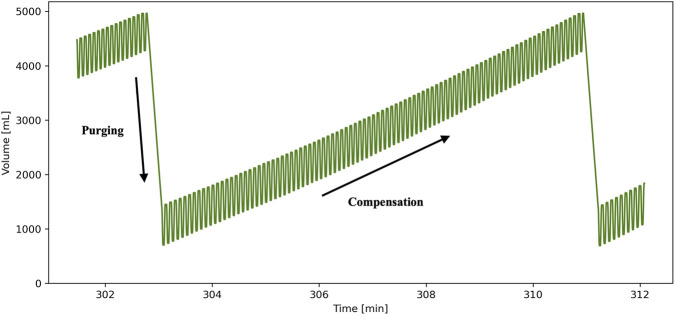
Evolution of the piston volume during ventilation of an *ex vivo* porcine lung with 450 mL/min dynamic leaks.

**FIGURE 3:**
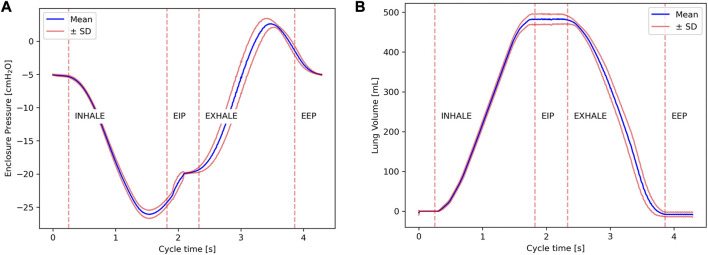
Normal ventilation of porcine lungs over 150 breaths with a 450 mL/min leak: **(A)** Mean pressure-time loop and **(B)** mean volume-time loop with respective standard deviations (SD).

At all times, during ordinary ventilation, whether in PCV or VCV, the motor angular targets described in the previous section are continuously re-calculated by means of a feedback loop. This loop uses the measured error between the desired (user-defined) and actual motor positions. These errors are fed into a Proportional-Integral (PI) controller, which constantly adjusts the piston targets. The resulting action of the piston when a leak is present is such that it must intake more volume to generate the same P_plat_ and output less volume not to surpass the set P_res_. Following this same logic, we can come to understand Figure 2 showing the ventilation of a pair of *ex vivo* porcine lungs using the PCV mode with P_res_ and P_plat_ targets respectively set to −5 and −20 cm H_2_O. Given the presence of a defect causing a moderate leak (450 mL/min), we can clearly see the progression of V_p_ over 150 respirations to keep input parameters relatively constant. Examining the superposition of all breaths temporally aligned using the cycle time started at the beginning of inhale (Figures 3A,B), we can appreciate how the piston reliably reaches the target pressures and volumes in spite of the compensation.

As one can expect, when the piston reaches its maximum physically allowed position, at which point it can no longer move back within its chamber, the system must purge out the accumulation of gas that was passively introduced in the enclosure environment. Exploiting the integrated pinch valves, the purging sequence is as follows: at the end of an inspiration, pinch A closes the airway shut and pinch B opens a piston outlet to the atmosphere; piston then moves forward which pushes most of its accumulated air directly out of the system until the homed position is reached; pinch B then closes and pinch A opens; ventilation resumes.

### 2.5 Leak quantification

Having now the ability to counteract the effect of leaks, it becomes necessary to quantify their magnitude. In fact, the system is equipped with two methods of quantifying the leak rate. The primary, similar to the commonly used digital thoracic drainage systems, provides a value of the leak rate calculated at a given pressure. This same process, which we have called static leak rate can be replicated using the piston motion to continuously equilibrate the pressure in the lung enclosure to a set value. Knowing the piston volume variation, we can then quantify the leak volume for a set pressure, given sufficient time is allowed for stabilisation (5 min). Owing to the reliance on enclosure pressure for ventilation, this method can only be utilised during ventilatory pause. A secondary method is instead based on the summation of actual volume compensated during the ventilation to maintain the input parameters as described above. Assuming we are working with similar pressures in regard to when the compensation is made, we can consider the volume delta corrected at each breath as the representation of the actual leak within that cycle. By computing the sum of compensated volumes over a given period divided by the duration of that period, we can derive the dynamic leak rate (mL/min). This of course is only valid when the system is considered stable, and the input settings remain unchanged. To validate performance of these features, the *Thopaz +* digitally monitored thoracic drainage system (Medela Healthcare, Baar, Switzerland) was used as a baseline due to its capacity to measure air leakage at different pleural pressure settings (Pompili et al., 2014).

### 2.6 Coughing

Coughing was implemented so that it can be triggered at any point during ventilation. Normally, for a single cough, the sequence can be summed up in three distinct phases: inspiratory phase, glottic closure and expiratory phase (Broaddus, 2016; McCool, 2006). In the first phase, a deep inspiration (DI) occurs, sometimes approaching vital capacity. The second phase starts once the epiglottis closes, during which the expiratory muscles contract to compress the inspired volume, generating subglottic pressures ranging from 40 to 400 cm H_2_O subglottic pressure in about 200 ms. The final phase comprises of the rapid opening of the epiglottis which in turn expulses part of the inhaled volume at a high flow rate (Broaddus, 2016). Endeavouring to follow the above-mentioned sequence, when triggered, the device sequentially performs five or six actions: initiation, DI, compression, expulsion, catch and re-initiate.

**FIGURE 4:**
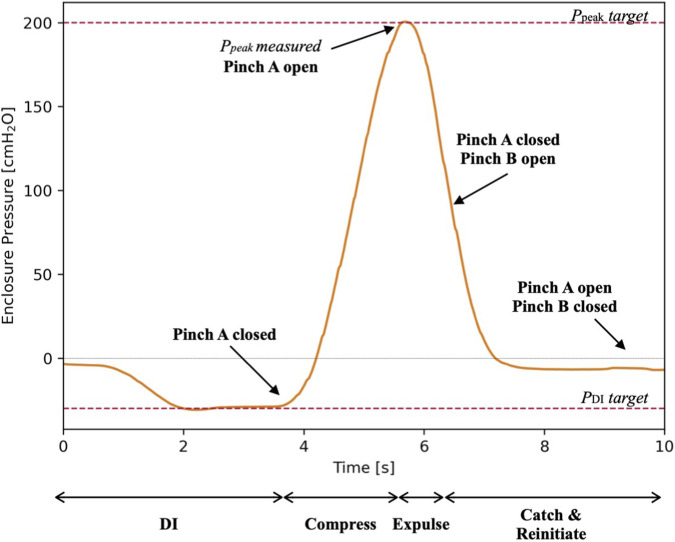
Plot of the enclosure pressure across time during a single cough with a P_peak_ target of 200 cm H_2_O and a P_DI_ target of −30 cm H_2_O.

After being triggered, to account for the dampening effect of the air interface and thus assure adequate compressive power, the device re-initiates its position to go further back if the piston volume is too low, allowing a more substantial potential volume delta. Once the piston is at an appropriate position, a DI is taken on the following breath by decreasing the target P_plat_ to the DI pressure (P_DI_) input by the user (i.e., −30 to −40 cmH_2_O). When this new P_plat_ is reached, pinch A (epiglottis) closes, and the device exhales with its maximum velocity and acceleration (i.e. 12 mm/s and 8 mm/s^2^), thus creating an increase in enclosure and airway pressures alike, until peak cough pressure (P_peak_) is reached, typically in the span of 1–2 s. It must be noted that this value was mainly imposed by technical limitations of our system, and is slower than physiological coughing. When reached, a timer is started, and pinch A momentarily opens, expulsing rapidly some of the inhaled air. After a predetermined time elapsed, on the order of 100–200 ms, pinch A is closed to “catch” the lungs and maintain some residual volume. To end the cough sequence, pinch B is open to re-equilibrate enclosure pressure allowing the piston to move back to its original position and resume ventilation. Though not explicitly needed when the lungs are in their proper thoracic cavity, the last two phases described are essential to avoid complete or partial lung collapse. Figure 4 illustrates this sequence, showing how the enclosure pressure progresses across the entire cycle of a standard cough with a target P_DI_ of −30 cm H_2_O and a target P_peak_ of 200 cm H_2_O.

### 2.7 Parameter calculation

To provide visibility on the lung state throughout experiments, parameters that can speak to the real-time elastance (E_L_) and resistance (R_L_) were generated using the widely exploited single compartment linear model. This mathematical model marked by its simplicity and parameter relevance, can be obtained using Equation 5 where P_tp_(t), V(t) and V̇(t) are respectively the transpulmonary pressure, the lung volume and lung flow over the laps of a breath. P_0_ is a constant acting as residual pressure assuring P_tp_(t) is nonzero when V(t) and V̇(t) are null (Bates, 2015).

$$P_{tp}=E_{L}V+R_{L}\dot{V}+P_{0}$$ (5)

With a least squared best fit of Equation 5 over a given period of time (i.e., a single breath), we can identify E_L_ and R_L_ with Equation 6 and Equation 7.

$$R_{L}=\frac{\sum_{i=1}^{N}V_{i}^{2}\sum_{i=1}^{N}\dot{V_{i}}P_{i}-\sum_{i=1}^{N}V_{i}\dot{V_{i}}\sum_{i=1}^{N}V_{i}P_{i}}{\sum_{i=1}^{N}V_{i}^{2}\sum_{i=1}^{N}\dot{V_{i}^{2}}-{\left(\sum_{i=1}^{N}V_{i}\dot{V_{i}}\right)}^{2}}$$ (6)

$$E_{L}=\frac{\sum_{i=1}^{N}V_{i}P_{i}-R_{L}\sum_{i=1}^{N}V_{i}\dot{V_{i}}}{\sum_{i=1}^{N}V_{i}^{2}}$$ (7)

Recognising the nonlinear variation in inhale and exhale dynamics due to hysteresis or other mechanisms (Bhana and Magan, 2023), the parameters have been separated accordingly.

### 2.8 Experimental protocol

**FIGURE 5:**
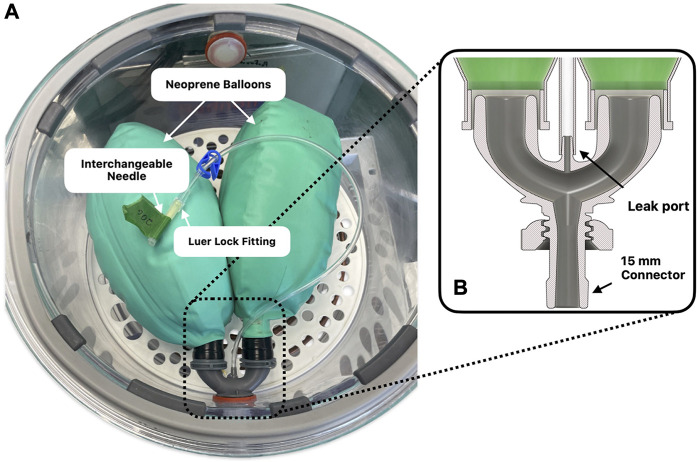
**(A)** Experimental set up of the double neoprene balloon equipped with a tube containing a Luer Lock fitting for the interchanging of needles of different gauges. **(B)** CAD model cross-section of the 3D printed connector.

This work being exploratory and emphasis having been put on versatility, validatory experiments where conducted using various sources. For its simplicity, ease of use and repeatability of conditions, a simulating apparatus was designed and built with an embedded controllable leak rate feature. The device consists of two neoprene balloons, commonly found in anesthesia circuits, fixed to a 3D printed connector (Figure 5). The connector in question had a dedicated leak port to which needles of various gauges (G) could be connected, to impose a specific effective open area and thus a specific leak rate. To that effect, seven configurations were studied: closed, 25, 23, 20, 16 and 14 G. To avoid erroneous dimensions furnished by the manufacture concerning the needles’ internal diameters (ID), cross-sections were measured using a digital microscope (26700-220; Aven Tools, Michigan, United States) and processed with the open-source image processing software ImageJ.

For consistent access to low-cost organs, lungs were sourced from a local slaughterhouse (Boucherie Bergeron, Coaticook, Canada) and immediately conserved at 4 °C after harvesting. Experiments were conducted the day after slaughter (i.e., within 36 h). The sex of the animals could not be retrieved, but was reported to mainly consist of females, the age of which ranged from 6 to 8 months, and the weight reported to be close to 90 kg. Due to major lacerations during harvesting or foam visible in the trachea caused by unintentional flooding during the scalding proceeding the animal’s slaughter, only 8 out of 30 organs procured were studied.

For the human model, all 3 pairs of lungs were obtained with informed consent following approval from our IRB (protocol 22.274). Two were obtained from patients with chronic obstructive disease (COPD) and one with idiopathic pulmonary fibrosis (IPF).

When working with *ex vivo* organs, preparation remained similar across experiments. After collection, thorough examination was performed to identify visible defects that could be fixed using surgical clamps or warrant discarding of the organ when they were too important. To bring the lungs up to temperature, they were placed in a hermetically sealed plastic bag and submerged in 37 °C–39 °C water using a common culinary immersion circulator. Heating time was varied depending on the initial temperature, aiming to commence experiment when above 32 °C (Moreno Garijo and Roscoe, 2020). An endotracheal tube (ETT) was then inflated in the trachea, and the specimen was placed on its dorsal side within the lung enclosure and connected to the airway opening port. For the specimens dissected too proximal to the lung during harvest, as was the case for the diseased human lungs to facilitate eventual transplant, a Penrose tube was sewn to the main bronchus to allow connection to an ETT. Once properly connected to the system, the lid was closed shut using a series of bar clamps and a preliminary assessment was done with a closed airway to ensure proper hermetic seal of the chamber. Recruitment was then accomplished in three stages to promote uniform parenchymal inflation without damaging the tissue. Firstly, a static pressure of −20 cm H_2_O was set and maintained within the enclosure for gradual alveolar expansion. Secondly, after a minute or so when recruitment visibly halted, the system upper and lower piston targets were configured as described previously, and PCV was activated. Finally, after a dozen breaths considered stable (i.e., when measured P_plat_ and P_res_ were repeatedly between ±0.5 cm H_2_O of set targets), a deep inspiration (DI) manoeuvre was induced, where the P_plat_ target was decreased to −30 cm H_2_O (P_DI_) for a single breath. Further DIs were performed when poor recruitment could be observed on the lung surface or if the tidal volume was well below the desired 6 mL/kg for human and 10 mL/kg for pig lungs (Aboelnazar et al., 2018). Once recruitment completed, normal ventilation was maintained without intervention for however long it was required.

To simulate leaks in a more clinically-relevant manner, the following protocol was applied. After recruitment and confirmation that the lungs were leak-free, ventilation was paused, and a thoracic surgeon member of the lab performed a wedge resection on the right upper lobe using a stapling system (60 mm, ECHELON™; Johnson and Johnson, New Jersey, United States). Next, ventilation was resumed, assuring once again proper recruitment. After 10 min of stable ventilation and two DIs of −30 cm H_2_O, three coughs were induced with a P_peak_ of 150, 200 then 300 cm H_2_O followed by a 10 min evaluation period between each. Dynamic leaks were monitored, and static leak was tested once ventilation was paused. Confirmation of leak detection and position thereof was conducted by inflating the lung with a positive pressure of 20 cm H_2_O and spraying soapy water on its surface. Due to the presence of surfactant in the soap, the formation of bubbles at the leak location allows easy visual confirmation. In the case of absence of leaks, lungs were ventilated without further intervention for an additional 6 h.

### 2.9 Data analysis

A record function enabled to passively store all sensor data and program variables on the computer’s hard drive at a 140 Hz frequency. Later, mathematical model implementation, data visualization, and signal processing was performed using Python 3.11 within a Jupyter Lab environment. All statistical analysis consisted of a 2-way ANOVA with Tukey’s multiple comparisons test performed on GraphPad Prism (Version 9.0.1; California, United States).

## 3 Results

Three distinct studies were conducted using the double neoprene balloon setup shown on Figure 5, and subsequently reproduced on either porcine or human lungs. The first study sought to evaluate the performance of the device in keeping ventilatory stability over time. The second study focused on evaluating air leak quantification using different developed features and a digital thoracic drainage system as reference standard. The third study focused on the evaluation of the coughing peak pressures and coughing integration to negative pressure ventilation. From the data gathered from each study, an additional analysis was conducted regarding the relevance of the biomechanics parameters extracted using the single compartment approximation.

### 3.1 Ventilation stability

Ventilation stability was studied across all leak configurations by using the double neoprene balloon setup on PCV then VCV modes. Data was acquired over 150 breaths each time a variable was changed to allow sufficient time for the system to reach a steady state. For moderate dynamic leaks (<1,000 mL/min), under PCV with a P_plat_ target of −20 cm H_2_O, we obtained a standard deviation of ±0.08 cm H_2_O centered on that target. In that experiment, though it was not controlled, V_t_ was measured to be 352.74 ± 3.15 mL. When switched to VCV, to remain consistent with the effort applied to the balloons, a V_t_ target of 350 mL was selected. Within the same leak range, we then obtained a standard deviation of ±2.1 mL. As both PCV and VCV used the same P_res_ of −5 cm H_2_O as a lower target, its standard deviation was categorised in the same group and was ±0.18 cm H_2_O. With the same target parameters, but for dynamic leaks considered severe (>1,000 mL/min), PCV yielded a standard deviation for P_plat_ of ±0.18 cm H_2_O, VCV yielded a standard deviation for V_t_ of ±11 mL and both methods yielded a standard deviation for P_res_ of ±0.38 cm H_2_O.

In PCV mode, when ventilating specimens with negligible to moderate dynamic leaks (<1,000 mL/min), the device consistently maintained standard deviation for P_plat_ and P_res_ respectively of ±0.64 cm H_2_O and ±0.47 cm H_2_O, for however long the experiment lasted (1–6 h). Figure 3 illustrates an example of the pressure-time and volume-time curves extracted from a porcine lung experiment with moderate leaks.

### 3.2 Leak assessment

The study was conducted using the same set-up and ventilatory parameters as described in the previous section. For each leak configurations, measurements were taken using the *Thopaz +* followed by the static leak function. Ventilation was then commenced to assess dynamic leaks with PCV then VCV. Across all methods, systems were given a 5 min period to stabilize prior to data acquisition. To ensure consistency in regard to the effort generated by the pressure on the specimen, static leak measurements for both the *Thopaz+* and the NPV system, were obtained using a target pressure of −13 cm H_2_O, as it represents the mean pressure it was subjected to in the breathing cycles. All experiments were conducted three times in all the different configurations (N = 3).

**FIGURE 6:**
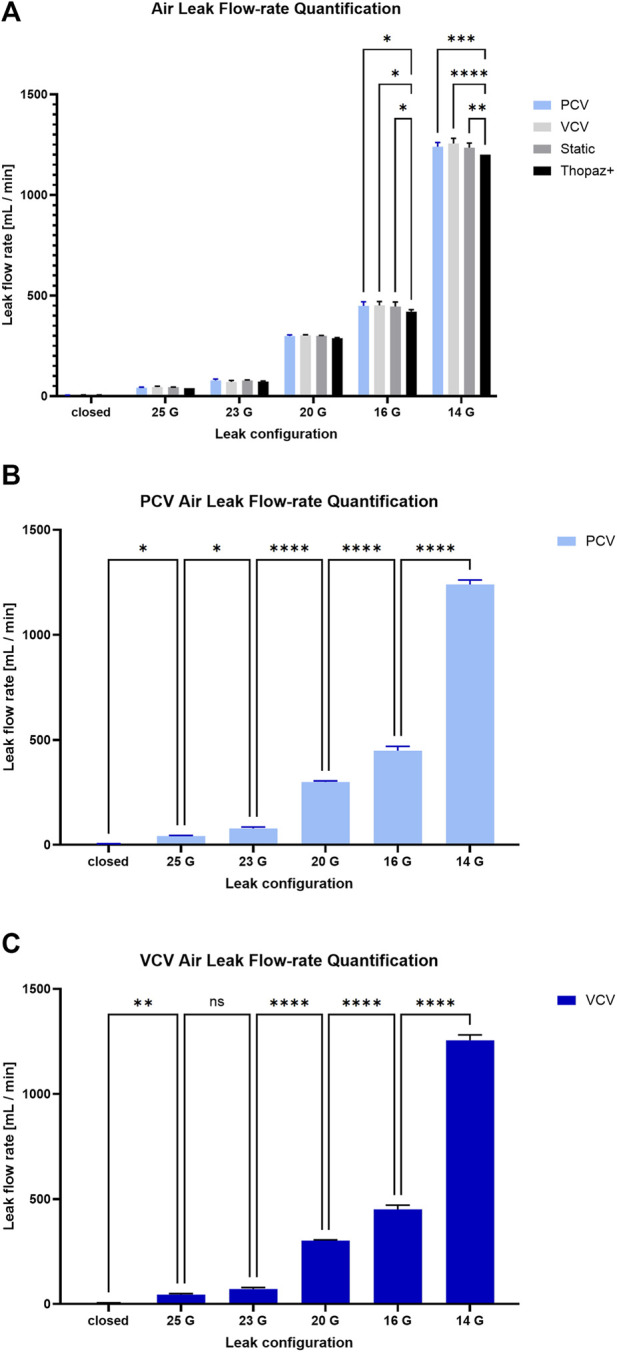
Results of the double neoprene balloon setup: **(A)** comparison of different methods of leak quantifications, **(B)** a close-up view of the dynamic leaks in the PCV mode for different leak configurations and **(C)** a close-up view of the dynamic leaks in the VCV mode for different leak configurations.

First, we sought to compare metrics-derived from our system (static, PCV and VCV) to the Thopaz + commercial system. As shown in Figure 6A, there were no significant differences between leak assessment methodologies for leak configurations ranging from entirely closed to 20 G. However, at 16 G, the flow rate estimated by the Thopaz + system was significantly lower than PCV (420.0 ± 10.0 mL/min vs. 449.0 ± 19.9 mL/min, p < 0.05), VCV (420.0 ± 10.0 mL/min vs. 451.0 ± 19.9 mL/min, p < 0.05) and static-derived (420.0 ± 10.0 mL/min vs. 445.3 ± 22.4 mL/min, p < 0.05) values, without significant differences between the three. Similarly, at 14 G, the flow rate estimated by the Thopaz + system was once again significantly lower than PCV (1,200.0 ± 0.0 mL/min vs. 1,239.7 ± 21.1 mL/min, p < 0.001), VCV (1,200.0 ± 0.0 mL/min vs. 1,255.0 ± 26.0 mL/min, p < 0.0001) and static-derived (1,200.0 ± 0.0 mL/min vs. 1,234.7 ± 23.0 mL/min, p < 0.01) values, still without significant differences between the three. However, since the Thopaz + reports highly discretized flow values, repeated measurements often return identical results, yielding an artificially low within group variance. As such, this limited resolution can overestimate statistical significance in ANOVA-based comparisons, and results involving the Thopaz + should therefore be interpreted with caution.

We then sought to compare the performance of the PCV and VCV parameters. As shown in Figures 6B,C, while the flow rates resulting from each leak configuration are significantly different for the PCV (p < 0.05), the VCV parameter is unable to differentiate between the air leak flow rates resulting from a 25 G and a 23 G leak configuration. This observation, combined with other practical factors, led us to elect PCV as the parameter of choice for air leak quantification.

Finally, three porcine lungs were dedicated to the air leak assessment in a more clinically comparable context. On the first two instances, dynamic leaks remained negligible (<20 mL/min) throughout the entirety of the experiment (7 h). On the third occasion, after the 300 cm H_2_O cough, a 90 mL/min dynamic leak was observed. After removal of the lid, leak was indeed confirmed to have originated from the staple line.

### 3.3 Cough simulation

Coughing was once again simulated using the double neoprene balloon setup in the closed configuration during regular PCV. The only variable in this experiment was the P_peak_ targeted in the different groups which were respectively fixed to 100, 150, 200, 250 then 300 cm H_2_O. Over the 150 coughs studied, 30 per group (N = 30), the standard deviation of the difference between the target and the measured ±1.30 cm H_2_O. In addition, after a completed cough sequence, the system took roughly 2 ± 1 breaths to return back to a stable ventilation. This delayed stability did not lead to conditions that would collapse the lung nor inflate it past its total lung capacity.

### 3.4 Parameter calculation

**FIGURE 7:**
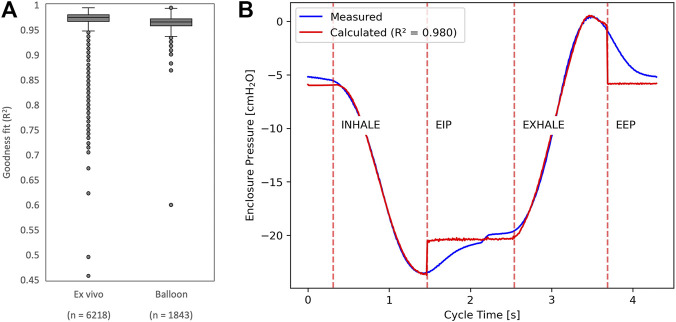
**(A)** Distribution of R^2^ values shown as a box-and-whisker plot, representing how well the parameter-driven model fit the measured data for all breaths obtained during *ex vivo* and double neoprene balloon experiments. **(B)** Pressure-time curve of a typical breath with the superposition of the measured and calculated curves.

The data obtained in the previous experiments were compiled for extrapolation of elastance and resistance parameters, to determine the ability for the chosen mathematical model to fit experimental measurements of pressure. The actual P_box_ readings for all breaths were superposed with the corresponding pressure calculated using the parameters in the single compartment approximation. R^2^ values were then generated for all breaths individually and plotted in Figure 7A. Over 1843 breaths (142 min) of ventilation with moderate to no leaks (<1,000 mL/min), R^2^ was for the greater part above 0.95. Likewise, when normally ventilating *ex vivo* specimens (4 porcine lungs and one lung from patient with IPF) with moderate to no leaks, across a total of 6,218 breaths (475 min), R^2^ was for the greater part above 0.95. In fact, Figure 7B illustrates a typical superposition of a single breath during the normal ventilation, where R^2^ is 0.98.

## 4 Discussion

In this work, we described a physiologically relevant *ex vivo* NPV model capable of convenient and reliable air leak assessment in lung explants. This NPV system was intentionally designed to aid in the better development of surgical devices related to lung resection surgery. Although several models have been proposed for NPV regarding small and large animal *ex vivo* lungs, our device improves upon these designs by avoiding certain shortcomings and tailoring the system to perform the best in the study of PAL, making it by design ideal for the research of air leak occurrence across a variety of procedures. As such, Table 1 lists and describes the different models and how they compare in some key features to the solution we proposed.

**TABLE 1:** Comparison of key ventilation features proposed in our Ex-PALM system and other relevant NPV models in the literature.

System features	Ex-PALM	Klassen et al. (2018)	Aboelnazar et al. (2018)	Wong et al. (2017)	Sattari et al. (2020)
Target application	PAL	PAL	Lung transplant	Respiratory mechanics	Respiratory mechanics
Pressure generation	Single piston	Single piston and mechanical ventilator	Dual ventilator	Single piston and Turbine	Dual piston
PEEP	No	Yes	Yes	No	Yes
Perfused	No	Yes	Yes	No	No
Air leak Compensation	Yes	Yes	-	No	No
Air leak Quantification	Yes	Yes	-	No	No
Fluid interface	Humid air	Saline	Humid air	Humid air	Air
Coughing	Yes	Yes	No	No	No

Although the model proposed by Klassen et al. initially appears to meet many of the previously outlined system requirements, its reliance on a liquid interface makes it a less suitable candidate for *ex vivo* PAL research. This approach limits access to the organs during ventilation and makes experimentation cumbersome and highly sensitive to poor lung quality. Although no such concerns were reported by the authors, it also remains unclear how the liquid would interact with the defect site throughout the experiment, as flooding from the enclosure fluid at the defect site is not inconceivable. This constraint may partly explain why the device has been used in only one study since its initial introduction (Eckert et al., 2018). Some of the key limitations restricting the utility of the other existing models in PAL research include the absence of leak compensation and quantification features, lack of cough simulation, and the requirement for Positive End-Expiratory Pressure (PEEP) to prevent lung collapse. The Ex-PALM system therefore tackles a critical unmet need by addressing several major limitations present in currently available models.

Other than perfusion, the most interesting feature which could be incorporated in future works is the characterisation of mechanical strain using digital image correlation (Mariano et al., 2020), as described by Sattari et al. (2020). Although not optimal in its described embodiment owing to the limited zones which can be studied (i.e., only the surface facing the camera) and the need to paint over the lung surface, a modified version could give insight on the tissue reactions responding to various induced ventilatory stresses. More importantly, knowing the strain dispersion post lung resection could provide greater understanding on the mechanical impact of staple lines on the surrounding tissue.

In both ventilation modes, the feedback loops enabled our device to maintain input parameters in a relatively stable manner despite presence of air leakage. Although only slight differences were observed between the two control modes when leaks were moderate, PCV was prioritized during *ex vivo* experimentation for its robustness. This was convenient when testing stapler performance, for example, where it was deemed more appropriate to remain consistent with the stress applied on the lung rather than to favor proper gas exchange. In VCV mode, especially considering specimens of varying sizes and conditions, the peak pressures may fluctuate and create inconsistent stress on the lungs, potentially compromising the validity of the air leakage findings. Be that as it may, VCV was nonetheless developed after observing a systematic decrease in the V_t_ over longer periods of time (>30 min) when ventilating *ex vivo*. In the future, though VCV may not be prioritised for the reasons listed above, a hybrid solution could be incorporated, where it is activated only when V_t_ falls below a certain threshold to inform the choice of P_plat_ in accordance with the new state of the lungs. As a matter of fact, regarding the decay in the measured V_t_ values, due to the short period in which it surfaced (<20 min), it can likely be linked to bronchoconstriction rather than tissue degradation. If PCV is needed, similar to clinical evidence, the lungs could be re-recruited with periodic DIs (Nadel and Tierney, 1961; Bendixen et al., 1964) directly integrated in normal ventilation (e.g., 8 DI/h).

Mainly, the significant differences observed with the Thopaz + system regarding air leak values in the 16 G and 14 G configuration were deemed acceptable. In addition to not necessarily being a proper ground truth, the Thopaz + system only provides 2 significant digits for the leak value displayed. This poor granularity repeatedly led to total absence of variance between independent experiments, ultimately resulting in more pronounced and statistically significant differences. Encouragingly, PCV exhibits said granularity, being able to differentiate between minute leak variations with relative ease; a promising result for the upcoming studies utilizing our methodology. To avoid the necessity of ventilatory pause, the more convenient dynamic leak was defined as the standard method of measurement for this device, although flow rate values may be validated with the static leak function. It must be noted that the performance of the leak quantification feature of the Ex-PALM system hinges on the assumption that compensated volume equals leak volume. Although our experimental data show a good match between the two, this match could potentially be compromised by inadequate system calibration. As such, regular quality control and calibration of the Ex-PALM system are essential for extensive experimental testing.

Clinically, persistent coughing is one of the most commonly encountered complications post lung surgery (Sawabata et al., 2005; Mu et al., 2023). Though there is no direct link between coughing and manifestation of pneumothoraces, the momentary severe intensification of strain on the lung tissue during a cough could exacerbate postoperative leaks. Importance was thus given to the implementation of this feature, following as closely as possible the usual dynamics of the event. Ultimately, the sequence remained similar in its essence but required some alterations to fit within our device’s capabilities. Mechanically, there were limitations regarding the maximum speed and acceleration, in turn compromising its ability to compensate for the dampened compression from the air interface. This did not impede on the capacity to reach the desired peak airway pressures, as was shown with the 150 simulated coughs on the double neoprene balloon setup. However, compression took about 10 times longer than physiologically expected and P_peak_ was not pushed all the way to the possible 400 cm H_2_O described in the literature (Broaddus, 2016). Although not ideal considering we may suppress the impact on the tissue of such a rapid and intense compression, we did not consider this a complete showstopper in of itself, as a relatively high peak stress on the lung is still reached. To that effect, coughing played an important role in the generation of leaks at the staple line during wedge resection experiments. Extrapolating to a clinical equivalent, this would be comparable to a case where surgeons, satisfied with the resection procedure and there seeming to be no apparent leaks, would close the patient up, later to find out the patient is unable to breathe on his own due to PAL and must remain hospitalised until healing occurs naturally.

While there are a variety of more exhaustive models that don’t limit themselves to the oversimplified single compartment representation of the lung, it is generally accepted that the non-linear effect of the tissue deformation does not play an important role in the pressure estimate for typical tidal breathing (Bates, 2015). As a matter of fact, for a given pair of lungs undergoing normal ventilation and with moderate leaks, we were able to predict the mean enclosure pressure using the calculated elastance and resistance parameters with excellent R^2^ values. Although these values are not necessarily meant to have clinical equivalence, since the entire thoracic structures aren’t represented in the current embodiment, it nonetheless gives room for inferring information about the state of the organs throughout an experiment. For example, this can be done by monitoring elastance over time for organ sourcing and protocol refinement, or eventually to link how certain mechanical properties affect the outcome of various experiments. This feature was shown to have some predictive significance but has not yet been exploited for any conclusions due to the lack of consistency in *ex vivo* data obtained so far.

The previously described performance assessments were validated, albeit in a less repeatable manner, using a limited amount of *ex vivo* human (N = 3) and animal specimens (N = 8). Qualitatively, there were no observable performance differences concerning the device in itself when comparing between the *ex vivo* organs and the double neoprene balloon setup. The main disparities lied more on the preparation, the initial state and the general evolution of the organ throughout time rather than how well the device performed the different actions responding to user inputs. In fact, the two COPD human lungs were excluded from the study due to poor quality of the organ, yielding in severe leaks from the get-go (<2000 mL/min). This brings up the question of eventual generalizability of an experimental protocols based on human specimens, as it is difficult account for the varying degrees of disease and condition in which they are obtained. Still, having been purposefully designed to accommodate samples of varying conditions, the Ex-PALM ventilation parameters can be tuned in a case-by-case manner to best suit the experimental objectives while continuously monitoring the mechanical response of the lungs.

When iterating on the protocol details, lung transplant research was used as a starting point and an ideal to strive towards (Watanabe et al., 2021; Buchko et al., 2020; Forgie et al., 2025). However, with less stringent requirements regarding the viability of the organ, we simplified steps where possible to avoid potential sources of error or unnecessary costs. Where this was most apparent is in the decision to avoid flushing and perfusing the lungs. Though it could have been interesting to delay tissue degradation, improper perfusion implementation can exacerbate oedema leading to inconsistencies in tissue mechanics. Although it was deemed outside the scope of the present work, the device was nonetheless designed with this capability in mind and is therefore equipped with dedicated ports appropriate for perfusate circulation. In the future, this feature could be exploited if a longer evaluation period is deemed pertinent. In addition, the obtained lungs were in unpredictable states and in conditions that were not always representative of the target population eligible or needing a lung resection. Nonetheless, they remain of great interest for general understanding of the diseases themselves and could be used as part of a more observational study with a smaller sample size to test devices in extreme conditions eventually allowing the extrapolation of other contributing factors leading to failures.

An important downside of this choice is maintaining the organs at a stable temperature. Not being able to benefit from the efficiency of dispersing thermal energy of the warm perfusate across the highly vascularised organ, it is more difficult to maintain a steady temperature. The heated body of water method was able to create an environment of maximum humidity and with temperatures ranging from 37 °C ± 2 °C for human specimens and 39 °C ± 2 °C for porcine specimens. However, this not only necessitated the organs to be preheated, but during procedures where the lid was open for longer periods of time, the temperature did drop considerably. As temperature has a known impact on mechanical properties of biological tissue, addressing temperature stability in our system is essential for larger scale studies. In the future, it would be beneficial to add an additional heating source, perhaps embedded in the enclosure lid, and to manipulate lungs exclusively through the dedicated working ports on the lid to limit temperature fluctuation and heat loss, with the added benefit of better simulating thoracoscopic procedures.

## 5 Conclusion

In this paper, we introduced an effective and intuitive clinically relevant platform for the evaluation and better understanding of PAL in an *ex vivo* setting. The accurate and repeatable dynamic evaluation of the lung state allows a seamless performance assessment, whether for products or surgical techniques, in an environment comparable to the spontaneous breathing of a patient. The added features of coughing, sighing and the capability of long-term ventilation, enables the user to put the lung through its paces while remaining within the expected physiological boundaries.

Future work includes application of the Ex-PALM device to investigating various PAL mitigation strategies, such as the comparison of commercialised endo staplers, the evaluation of lung sealant solutions and the development of intra-operative air leak localisation methods, to name a few. Moreover, the Ex-PALM can be applied to research avenues unrelated to PAL treatment, for example, to the study of bronchoscopic procedures or as a training platform where NPV simulates a non-intubated patient.

## Data Availability

The raw data supporting the conclusions of this article will be made available by the authors, without undue reservation.
